# Impact of nicotine pathway downregulation on polyamine biosynthesis and leaf ripening in tobacco

**DOI:** 10.1002/pld3.329

**Published:** 2021-05-27

**Authors:** Greta Nölke, Ivana Chudobova, Marcel Houdelet, Daniel Volke, Marcos Lusso, Jesse Frederick, Chengalrayan Kudithipudi, Yanxin Shen, Ujwala Warek, James A. Strickland, Dongmei Xu, Helga Schinkel, Stefan Schillberg

**Affiliations:** ^1^ Fraunhofer Institute for Molecular Biology and Applied Ecology IME Aachen Germany; ^2^ Research Development & Regulatory Affairs Altria Client Services LLC Richmond VA USA

**Keywords:** polyamines, inhibition of nicotine biosynthesis, PR50, putrescine methyl transferase, ornithine decarboxylase, ripening markers

## Abstract

Traditional breeding and molecular approaches have been used to develop tobacco varieties with reduced nicotine and secondary alkaloid levels. However, available low‐alkaloid tobacco varieties have impaired leaf quality likely due to the metabolic consequences of nicotine biosynthesis downregulation. Recently, we found evidence that the unbalanced crosstalk between nicotine and polyamine pathways is involved in impaired leaf ripening of a low‐alkaloid (LA) Burley 21 line having a mutation at the *Nic1* and *Nic2* loci, key biosynthetic regulators of nicotine biosynthesis. Since the *Nic1* and *Nic2* loci are comprised of several genes, all phenotypic changes seen in LA Burley 21 could be due to a mixture of genetics‐based responses. Here, we investigated the commercial burley variety TN90 LC and its transgenic versions with only one downregulated gene, either putrescine methyl transferase (PMT‐RNAi) or PR50‐protein (PR50‐RNAi). Nicotine levels of cured lamina of TN90 LC, TN90 PMT‐RNAi and TN90 PR50‐RNAi, were 70.5 ± 3.8, 2.4 ± 0.5, and 6.0 ± 1.1 mg/g dry weight, respectively. Low‐alkaloid transgenic lines showed delayed leaf maturation and impaired leaf quality. We analyzed polyamine contents and ripening markers in wild‐type TN90 control plants (WT) and the two transgenic lines. The ripening markers revealed that the PMT‐RNAi line showed the most pronounced impaired leaf maturation phenotype at harvest, characterized by higher chlorophyll (19%) and glucose (173%) contents and more leaf mesophyll cells per area (25%), while the ripening markers revealed that maturation of PR50‐RNAi plants was intermediate between PMT‐RNAi and WT lines. Comparative polyamine analyses showed an increase in free and conjugated polyamines in roots of both transgenic lines, this being most pronounced in the PMT‐RNAi plants. For PMT‐RNAi plants, there were further perturbations of polyamine content in the leaves, which mirrored the general phenotype, as PR50‐RNAi transgenic plants looked more similar to the WT than PMT‐RNAi transgenic plants. Activity of ornithine decarboxylase, the enzyme that catalyzes the committing step of polyamine biosynthesis, was significantly higher in roots and mature leaves of PMT‐RNAi plants in comparison to WT, while there was no increase observed for arginine decarboxylase. Treatment of both transgenic lines with polyamine biosynthesis inhibitors decreased the polyamine content and ameliorated the phenotype, confirming the intricate interplay of polyamine and nicotine biosynthesis in tobacco and the influence of this interplay on leaf ripening.

## 1 INTRODUCTION

Extensive research based on traditional breeding and molecular biology approaches has been used to better understand the nicotine biosynthetic pathway in tobacco plants, how this pathway is regulated and the role that nicotine has in the plant. Reducing nicotine and related alkaloid levels has been a breeding target for tobacco scientists, and tobacco breeding lines with low‐alkaloid content have been around for several decades (Legg et al., 1970). These plants were generated through traditional breeding and possess recessive alleles at the *Nic1* and *Nic2* loci (sometimes also named A and B loci), which have a synergetic effect of downregulating nicotine biosynthesis (Kidd et al., 2006; Legg & Collins, 1971; Shoji et al., 2010). Unfortunately, the leaf ripening process is disturbed in these low‐alkaloid (LA) tobacco varieties leading to inferior leaf quality (Chaplin & Burk, 1983; Chaplin & Weeks, 1976; Legg et al., 1970). Recently, we showed that the crosstalk between nicotine and polyamine biosynthesis is disturbed in these plants, and the accumulation of free and conjugated polyamines contributes to the impairment of leaf ripening (Nölke et al., 2018). Higher levels of polyamines have been shown to increase the longevity of tomato vines and to delay ripening and leaf senescence in transgenic tomato and salad plants (Mehta et al., 2002; Nambeesan et al., 2010; Serafini‐Fracassini et al., 2010). Polyamines may act directly by stabilizing cell walls or through crosstalk with phytohormones such as ethylene, abscisic acid, cytokines, and gibberellins (Anwar et al., 2015). Treatment of LA Burley 21 plants with polyamine biosynthesis inhibitors or ethephon (Nölke et al., 2018) reduced the accumulation of polyamines and achieved a partial amelioration of the aberrant phenotype of the LA Burley 21. However, there is a series of genes (≥8) deleted in LA Burley 21, relative to Burley 21 (Shoji & Hashimoto, 2015; Shoji et al., 2010), and some of these genes that are not related to nicotine biosynthesis could also have an influence on the general physiology, stress response, and senescence of the tobacco plants (Chaplin & Weeks, 1976; Kidd et al., 2006). This makes it difficult to study the effect that the overly accumulating polyamines have on the ripening process in LA Burley 21 tobacco. Therefore, evaluating polyamines in transgenic plants, ones that have low‐alkaloid content due to the downregulation of only one enzyme in the nicotine biosynthesis pathway, should give a clearer picture of the influence that polyamines have on the ripening process of LA tobacco.

There have been various transgenic approaches to reduce alkaloids in tobacco, e.g. downregulation of ornithine decarboxylase (ODC) (Dalton et al., 2016; DeBoer et al., 2013), berberine bridge enzyme‐like (BBL) (Lewis et al., 2015) or putrescine methyltransferase (PMT) (Chintapakorn & Hamill, 2007) enzymes. While all of these strategies lead to reduced alkaloid content, most have drawbacks; for example, the plants with the downregulated ODC had not only lower alkaloid content but also lower polyamine content leading to a dwarfed phenotype (Nölke et al., 2005), while downregulation of the *BBL* gene family resulted in reduced yields of cured tobacco (Lewis et al., 2015). Thus, it seems that the ideal process by which LA tobacco varieties could be developed and used for commercial manufacture of LA tobacco products of high quality has still not been found.

Here, we describe our analysis of two transgenic Burley TN90 plants, the PMT‐RNAi and PR50‐RNAi plants (Kudithipudi & Hayes, 2018; Wang et al., 2000), which have a suppressed PMT gene or PR50 gene via RNA interference, resulting in lower nicotine content. We conducted greenhouse experiments to compare the phenotype of these plants with WT plants using ripening markers (Nölke et al., 2018) and analyzed the polyamine content in the roots and leaves of both the transgenic and the WT plants during the ripening process. For PMT‐RNAi plants, the activity of ODC and arginine decarboxylase (ADC), as the enzymes responsible for polyamine formation, was investigated. The transgenic plants were furthermore treated with the polyamine inhibitor difluoromethylornithine (DFMO) to study if such treatment has an influence on the ripening phenotype of the plants. The data generated here give further insights into the relationship among nicotine biosynthesis, polyamine levels, and leaf/plant morphology.

## 2 RESULTS

### 2.1 Morphological and biochemical differences between low‐alkaloid tobacco varieties and WT during leaf ripening

**FIGURE 1:**
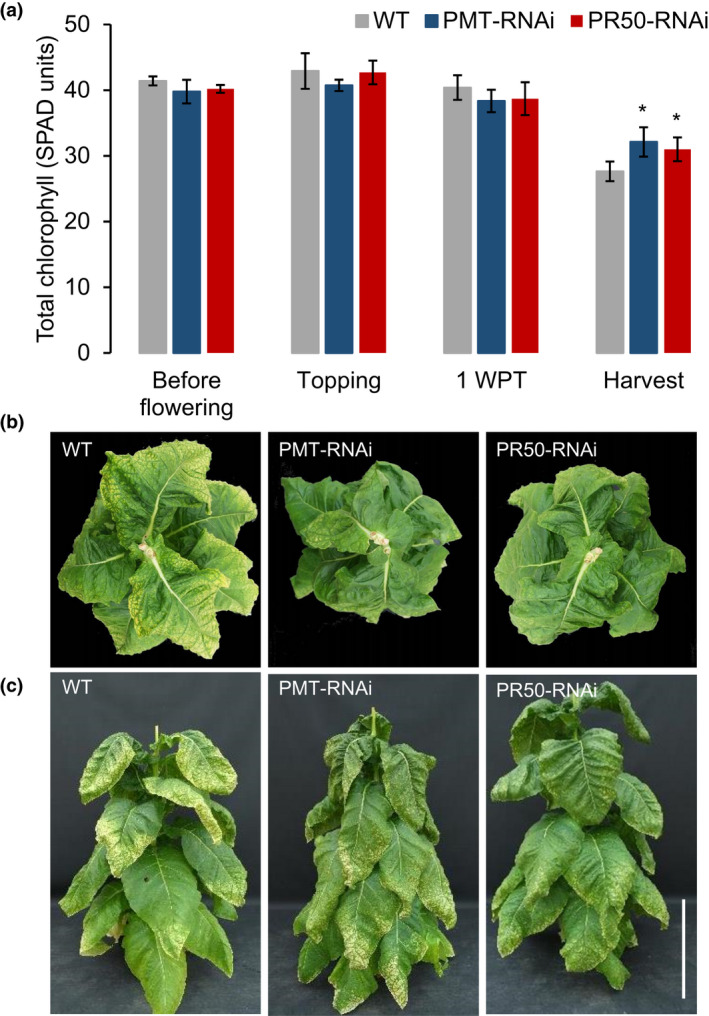
Phenotype characterization of TN90 WT, PMT‐RNAi, and PR50‐RNAi transgenic tobacco plants. (a) Total chlorophyll content in TN90 WT and transgenic PMT‐RNAi and PR50‐RNAi lines. Chlorophyll was measured twice in all leaves bigger than 15 cm in length at different developmental stages. Values are given as mean of four plants measured in above‐described way. Error bars represent standard deviation of the mean. Statistical difference from WT is shown: *p < .05. (b and c) Phenotype of representative WT, PMT‐RNAi, and PR50‐RNAi plants at harvest. Bar =50 cm

To evaluate the progression of senescence in the PMT‐RNAi, PR50‐RNAi, and WT lines, we monitored the total chlorophyll content in all fully expanded leaves at different stages of plant development. Chlorophyll levels were decreased in all three plant lines at harvest compared to the other three time points (before flowering, at topping, and 1 week post‐topping) (Figure 1a). However, the leaves of the PMT‐RNAi and PR50‐RNAi plants contained significantly (*p* < .05) higher levels of chlorophyll than the WT at harvest (16% and 12% more in the PMT‐RNAi and PR50‐RNAi, respectively). Consequently, the leaves of PMT‐RNAi and PR50‐RNAi plants were greener than the WT (Figure 1b,c).

**FIGURE 2:**
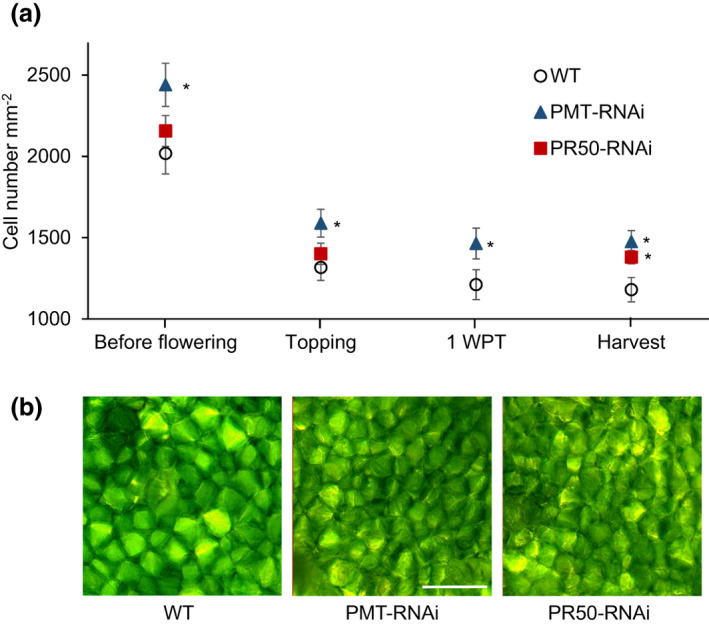
Analysis of mesophyll cell number in WT and transgenic lines. (a) Time‐course evaluation of mesophyll cell number in leaf 15. Values are given as mean of six biological replicates. Error bars represent standard deviation of the mean. Statistical difference from WT is shown: *p < .05. Data for PR50‐RNAi line at 1 WPT not measured. (b) Representative photos of WT, PMT‐RNAi, and PR50‐RNAi leaf mesophyll cells in leaf 15 at 1 WPT. Bar = 100 µm. BF, before flowering; WPT, week post‐topping

Evaluation of the size of the mesophyll cells in leaf 15 (numbered from base of the plant) revealed that the PMT‐RNAi plants had smaller cells compared to wild type (Figure 2b). The number of cells per mm^2^ leaf area was higher throughout the leaf ripening ranging from 21% more (before flowering, at topping, and 1 week post‐topping (WPT)) to 25% more (at harvest) compared to the WT (Figure 2a). The PR50‐RNAi plants contained 17% more mesophyll cells per mm^2^ leaf area at harvest, but no significant difference from the WT was measured at earlier time points (Figure 2a).

Monitoring of the free glucose concentration in leaf 11 (numbered from base of the plant) showed that both transgenic lines had significantly (*p* < .05) more glucose than the WT at topping (140% in PMT‐RNAi line and 42% in PR50‐RNAi) (Table 1). The glucose content in PMT‐RNAi increased more than in the WT during the whole leaf ripening process, reaching the highest levels at harvest (173% more than in WT). No glucose measurements were performed for PR50‐RNAi after topping.

**TABLE 1:** Analysis of glucose content (mg/L) in leaves of wild‐type and transgenic tobacco lines at different stages of plant development. WPT: week post‐topping

Lines	Before flowering	Topping	1 WPT	Harvest
WT	262.5 ± 21	214.7 ± 12.5	769.9 ± 58.8	3182.5 ± 540
PMT‐RNAi	256.4 ± 37	515.3 ± 73*	1,134 ± 79.3*	8698.9 ± 650*
PR50‐RNAi	260.1 ± 26	304.7 ± 22*	n.m.	n.m.

Note

Values represent means ± *SD* for leaf 11 (*n* = 4).

Abbreviation: n.m., not measured.

* *p* < .05.

### 2.2 Both transgenic genotypes accumulated higher levels of polyamines in roots

**FIGURE 3:**
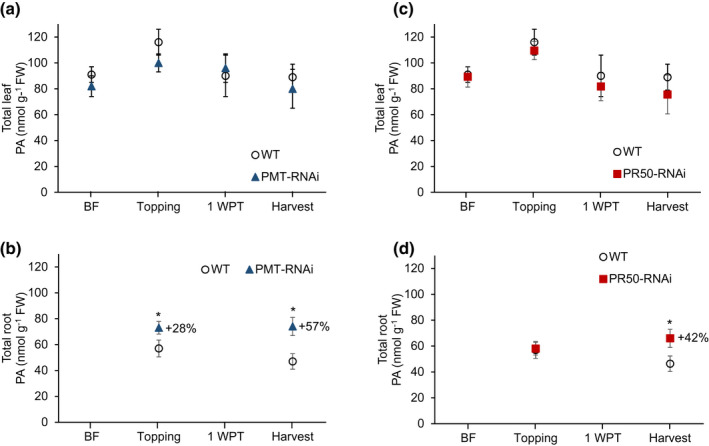
Time‐course monitoring of total polyamine content in WT and transgenic plants. Total polyamine content in leaves of PMT‐RNAi (a) and PR50‐RNAi (c). Leaf samples were collected from leaves at the same stage of development: before flowering (leaf 12), at topping (leaf 19), 1 WPT (leaf 22), and at harvest (leaf 24). Total polyamine content in roots of PMT‐RNAi (b) and PR50‐RNAi (d). Root samples were collected at topping and harvest. Values are given as mean of eight (a, c, d)/six (b) biological replicates. Error bars represent standard deviation of the mean. Statistical difference from WT is shown: *p < .05. 1 WPT: 1 week post‐topping; BF, before flowering; FW, fresh weight; PA, polyamine

To investigate the impact of the PMT and PR50 downregulation on polyamine biosynthesis, we analyzed the levels of free and conjugated putrescine, spermidine, and spermine by liquid chromatography tandem mass spectrometry (LC‐MS/MS). Time‐course monitoring of the total polyamine content in leaves at the same developmental stage—that is, leaf 12 before flowering, leaf 19 at topping, leaf 22 at 1 WPT, and leaf 24 at harvest—revealed no significant differences in polyamine levels in PMT‐RNAi and PR50‐RNAi plants compared to WT (Figure 3a,c). In contrast, polyamine analysis in roots showed that the PMT‐RNAi plants accumulated significantly (*p* < .05) higher levels of total polyamines at topping (28%) and at harvest (57%), compared to the WT (Figure 3b). Significantly (*p* < .05) higher total polyamine content was also measured in the roots of PR50‐RNAi plants at harvest (42%) than in the WT (Figure 3d).

**FIGURE 4:**
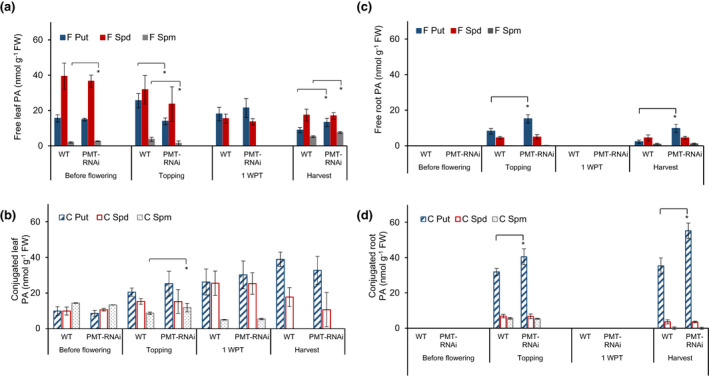
Polyamine content in leaves and roots of WT and PMT‐RNAi TN90 plants. Free (F) and conjugated (C) putrescine (Put), spermidine (Spd), and spermine (Spm) fractions in leaves (a, b) and roots (c, d) of WT and PMT‐RNAi TN90 plants before flowering (leaf 12), at topping (leaf 19, roots), 1 WPT (leaf 22), and at harvest (leaf 24, roots). Samples were collected 6 hr after illumination, frozen immediately in liquid nitrogen, and analyzed by HPLC. Values are given as mean of four biological replicates. Error bars represent standard deviation of the mean. Statistical difference is shown: *p < .05, indicating that the transgenic PMT‐RNAi TN90 line is significantly different from the WT TN90. Only samples from topping and harvest were available for roots. 1 WPT, 1 week post‐topping; FW, fresh weight

**FIGURE 5:**
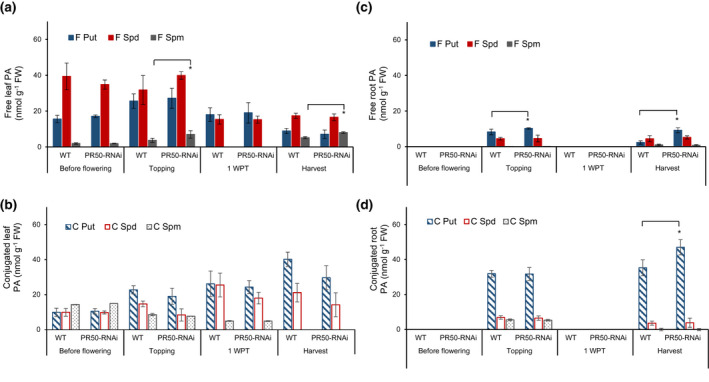
Polyamine content in leaves and roots of WT and PR50‐RNAi TN90 plants. Free (F) and conjugated (C) putrescine (Put), spermidine (Spd), and spermine fractions (Spm) in leaves (a, b) and roots (c, d) of WT and PMT‐RNAi and WT plants before flowering (leaf 12), at topping (leaf 19, roots), 1 WPT (leaf 22), and at harvest (leaf 24, roots). Samples were collected 6 hr after illumination started, frozen immediately in liquid nitrogen, and analyzed by HPLC. Values are given as mean of four biological replicates. Error bars represent standard deviation of the mean. Statistical difference is shown: *p < .05. 1WPT, 1 week post topping; FW, fresh weight

Comparative analysis of the polyamine composition revealed that before flowering PMT‐RNAi leaves contained significantly (*p* < .05) higher free spermine content (40%), while no changes in other polyamine fractions were observed (Figure 4a,b). Conversely, at topping the levels of free putrescine and spermine decreased by 46% and 62%, respectively, while the conjugated spermine increased by 73% (Figure 4a,b). No significant difference in the polyamine composition was observed at 1 week post‐topping, and at harvest the free putrescine and spermine content increased significantly (*p* < .05) by 34% and 54%, respectively, compared to the WT. Free spermine was significantly higher in the leaves of PR50‐RNAi line at topping and at harvest (90% and 54% higher, respectively), while no other differences in the polyamine composition compared to the WT were measured in the leaves of the PR50‐RNAi plants (Figure 5a,b).

In the roots, the free putrescine fractions increased during ripening of the PMT‐RNAi and PR50‐RNAi plants compared to the WT. The greatest relative increase in free putrescine was observed at harvest, that is, 320% increase in PMT‐RNAi (Figure 4c) and 296% increase in PR50‐RNAi lines (Figure 5c). The PMT‐RNAi lines had significantly (*p* < .05) higher conjugated putrescine at topping (27%) and harvest (56%) (Figure 4d). Conjugated putrescine was also significantly higher in the roots of PR50‐RNAi line at harvest (33%) compared to the TN90 control (Figure 5d). Taken together, these data indicate that the PMT downregulation has a stronger impact on the polyamine biosynthesis pathway than the PR50 downregulation.

### 2.3 Enzymatic activity of ODC varies significantly between PMT‐RNAi and WT

**FIGURE 6:**
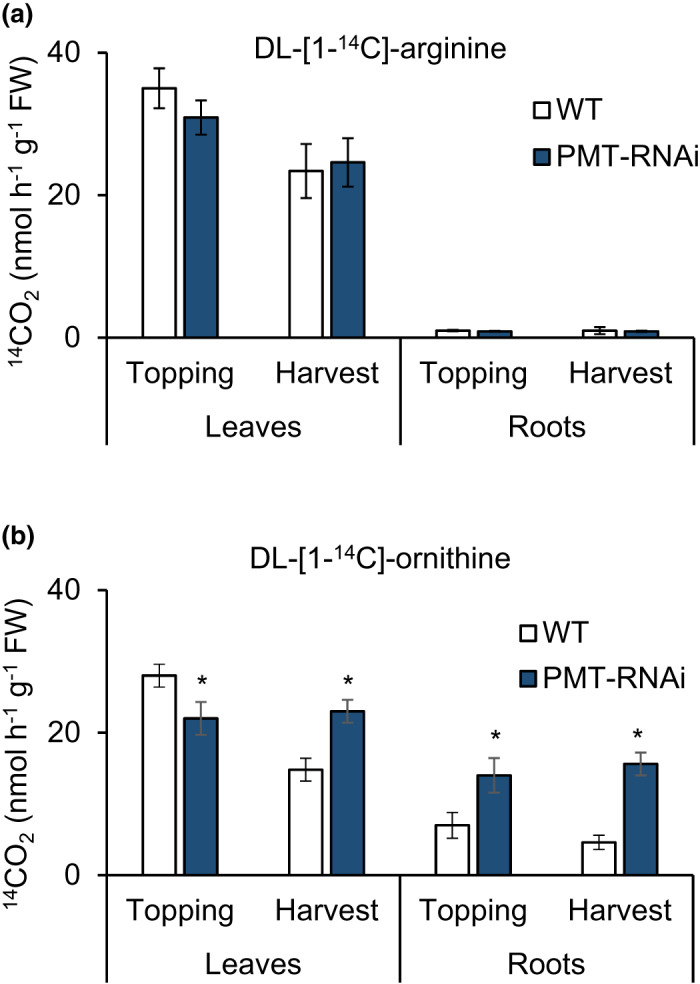
Activity of polyamine biosynthesis enzymes. Analysis of arginine decarboxylase (ADC) (a) and ornithine decarboxylase (ODC) (b) activity in leaves and roots of WT and PMT‐RNAi plants at topping (leaf 23, roots) and harvest (leaf 23, roots). Values are means of three biological replicates. Error bars represent standard deviations of the mean. Statistical difference from WT is shown: *p < .05

To further investigate the effect of PMT downregulation on polyamine biosynthesis, the activities of ADC and ODC were analyzed in the leaves and roots of the PMT‐RNAi plants that showed the strongest changes in the polyamine content, and the WT plants as well (Figure 6). The ODC activity was significantly lower (−22%, *p* < .05) in the leaves of PMT‐RNAi plants at topping, but was significantly higher (55%, *p* < .05) in leaves at harvest. Roots of the PMT‐RNAi plants showed significantly higher ODC activity both at topping (100%, *p* < .05) and at harvest (239%, *p* < .05). While in roots the ADC activity remained low in accordance with earlier findings (Nölke et al., 2018), in leaves ADC activity followed the same pattern as ODC activity, although the differences between PMT‐RNAi and WT were less pronounced and not statistically significant.

### 2.4 Inhibition of polyamine biosynthesis mitigates PMT‐RNAi phenotype

Given the evidence of enhanced ODC activity and higher polyamine levels as well as the undesirable leaf morphology at harvest in PMT‐RNAi plants, we evaluated the effect that treatment with DFMO, an irreversible inhibitor of ODC, had on the PMT‐RNAi and PR50‐RNAi plants. The treatment with DFMO started directly after topping and continued for 4 weeks until harvest.

**FIGURE 7:**
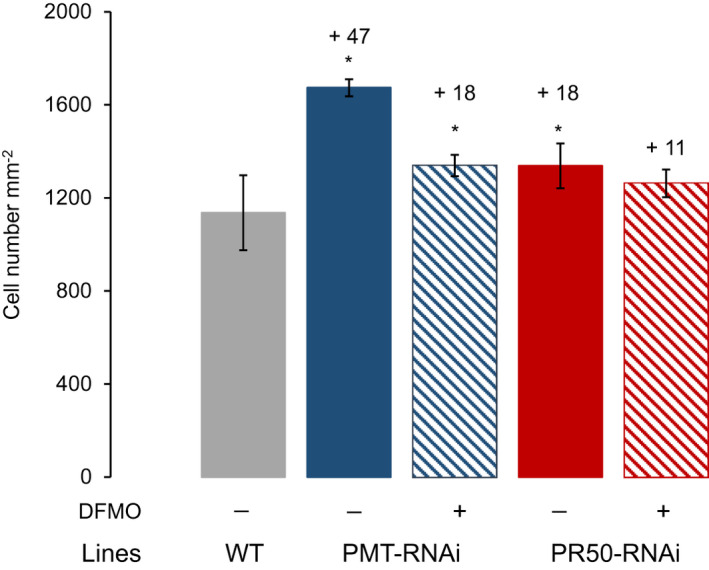
Analysis of mesophyll cell number in WT, PMT‐RNAi, and PR50‐RNAi untreated (−) and treated (+) with DFMO. Mesophyll cell numbers were determined at harvest in leaf 25. Values are given as mean of six biological replicates. Error bars represent standard deviation of the mean. Statistical difference from WT is shown: *p < .05

The DFMO treatment achieved a partial amelioration of the morphological phenotype, that is, the leaves of the PMT‐RNAi plants at harvest took on some of the characteristics of the WT leaves. Similarly, evaluation of the mesophyll cells showed that DFMO treatment led to a significant reversion of the mesophyll cell number in PMT‐RNAi (from 47% to 18%; *p* < .05). Although not significantly different, a similar tendency was also observed for PR50‐RNAi plants (from 18% to 11%) at harvest (Figure 7).

**FIGURE 8:**
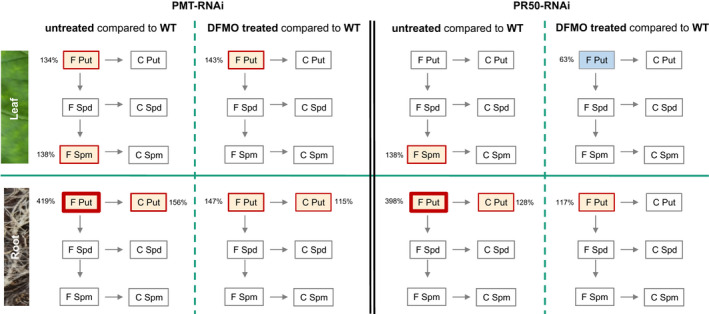
Comparative analysis of free and conjugated polyamine content in leaves and roots of WT, PMT‐RNAi, and PR50‐RNAi untreated and treated plants with polyamine biosynthesis inhibitor DFMO at harvest. WT, PMT‐RNAi, and PR50‐RNAi plants were grown in the greenhouse in the absence (WT, PMT‐RNAi, and PR50‐RNAi) or presence (PMT‐RNAi and PR50‐RNAi) of polyamine biosynthesis inhibitor DFMO (2 mM). Treatment was performed three times per week from topping to harvest for a period of 4 weeks. Samples were collected from leaf 23 or roots of four biological replicates per genotype or treatment. The ratio between polyamine content from untreated or DFMO‐treated PMT‐RNAi (1. and 2. panel from left) or PR50‐RNAi plants (3. and 4. panel) and WT is given in percent. Grey: No significant difference from the WT (=100%); red frame: significantly (*p* < .05) higher amount of polyamines compared to WT (>100%), the thickness of the box frames depicts the magnitude of the increase; and blue background: significantly (*p* < .05) lower amount of polyamines compared to WT (<100%)

The analysis of polyamine levels revealed that DFMO treatment led to a reduction in free and conjugated putrescine and free spermine in PMT‐RNAi and PR50‐RNAi plants (Figure 8). This reversing effect of the DFMO on the polyamine biosynthesis was most pronounced in the roots, resulting in reduction in free putrescine in PMT‐RNAi from 419% (PMT‐RNAi compared to WT) to 147% (DFMO‐treated PMT‐RNAi compared to WT) and in PR50‐RNAi from 398% (PR50‐RNAi compared to WT) to 117% (DFMO‐treated PR50‐RNAi compared to WT), while the reduction in the conjugated putescine was from 156% (PMT‐RNAi compared to WT) to 115% (DFMO‐treated PMT‐RNAi compared to WT) and from 128% (PR50‐RNAi compared to WT) to the level of the WT plants (100%; DFMO‐treated PR50‐RNAi compared to WT). A similar reversing effect was also observed in leaves with a decrease in free spermidine to the level of the WT plants.

## 3 DISCUSSION

Low nicotine content tobacco has the side‐effect of aberrant leaf maturation which in turn leads to inferior product quality. As analyzed previously (Nölke et al., 2018), polyamine biosynthesis is disturbed and upregulated in LA Burley 21 tobacco, which has lost eight or more genes, making analyses of cause–effect relationships difficult. In this study, we investigated two different transgenic TN90 plant lines where each has only one directly downregulated gene, either PMT (a key enzyme in nicotine biosynthesis) or PR50 (a 40S ribosomal protein S 12 homolog that is differentially expressed in roots of *Nicotiana tabacum* cv Burley 21 during the early stages of alkaloid biosynthesis) (Wang et al. 2000). Downregulation of PMT or PR50 genes led to lower nicotine levels in tobacco (Kudithipudi & Hayes, 2018). This work was conducted to provide insights into how the leaf ripening process is disturbed in these low‐alkaloid (LA) tobacco varieties leading to inferior leaf quality and gain a better understanding of the interrelationship between polyamines and the nicotine biosynthetic pathways.

The phenotype of the transgenic plants was compared to WT plants grown under the same cultivation conditions in the greenhouse. Using the ripening markers that we established before (Nölke et al., 2018), we confirmed that the leaf maturation was disturbed in these lines as the transgenic plants had significantly higher chlorophyll contents than the WT plants at harvest as well as a higher number of leaf mesophyll cells per leaf area. For both markers, PMT‐RNAi plants showed a stronger deviation from the WT phenotype than PR50‐RNAi plants which fits well with the observation of overall leaf quality of the PMT‐RNAi line being poorer than the quality of PR50‐RNAi line (Kudithipudi & Hayes, 2018).

The glucose concentrations in the PMT‐RNAi leaves increased more rapidly and to higher values than in the WT so that the PMT‐RNAi plants contain nearly three times as much glucose compared to the WT at harvest. Burley tobacco is characterized by low sugar concentrations in the leaves (Banožić et al., 2020) so an increase in glucose presents a potentially negative influence on the tobacco quality. It is known that while higher sugar concentration often increases the quality of a tobacco, there needs to be a balance among sugars, waxes, and resins (Mendell et al., 1984). It can be assumed that the inferior product quality of PMT‐RNAi tobacco is partially due to the high glucose content in the leaves.

When total polyamines were measured in the transgenic and the WT plants, no significant differences were detected in leaves, while roots showed a significant increase in polyamines in the PMT‐RNAi and PR50‐RNAi plants compared to WT. The increase in total polyamines is more distinct in PMT‐RNAi roots than in PR50‐RNAi roots, which fits well with the data of the phenotypic ripening markers. A similar connection between a suppressed PMT gene and increased polyamines has been reported for *Hyoscyamus niger* (Geng et al., 2018). The higher concentration of polyamines in roots of the transgenic plants compared to roots of the WT became more distinct from topping to harvest, that is, during the leaf maturing process. Senescence that is induced by topping is developing normal in the WT but in the transgenic plants this senescence is counteracted by the higher concentrations of polyamines, leading to the aberrant phenotype with higher chlorophyll contents and smaller cells in the leaves.

Putrescine is both the precursor of other polyamines and the substrate of PMT, and thus also a precursor for alkaloids (Wang et al. 2000). As both ADC and ODC catalyze putrescine production, we checked the activities of these enzymes in PMT‐RNAi plants. At harvest, ODC activity is significantly higher in both leaves and roots of PMT‐RNAi plants compared to the WT; for ADC, the trend is the same in leaves while there is very little activity in roots which is not unexpected, as ADC is mainly present in aerial parts of the plant (Bortolotti et al., 2004). The pattern seen for ODC activity fits well with other data that we collected; polyamines accumulate over time and mostly in roots. It seems illogical that the decrease in nicotine and the concomitant increase in polyamines happens in the roots, while the effects as a retarded senescence are seen in the leaves, however, similar effects have been observed before (Ruiz, Rios, et al., 2006; Ruiz, Rivero, et al., 2006; Sato et al. 2001) and it is known that polyamines are translocated within the plant from the roots to the upper parts and vice versa (Beraud et al. 1992; Caffaro et al. 1993; Rabiti et al. 1989).

The polyamine content in plants is tightly controlled, and previous studies have shown that conjugation of polyamines with biomacromolecules, for example, proteins, membranes, lignin, or hydroxycinnamic acid, is one of the mechanisms plants use to control the intracellular free polyamine content (Bassard et al., 2010; Nölke et al., 2018; Torras‐Claveria et al., 2012). Accordingly, PMT‐RNAi and PR50‐RNAi plants with increased polyamines also show increased polyamine conjugation. There was an increase in conjugated polyamines with age in both leaves and roots in the WT and in the two transgenic lines, but it was in roots that a significant difference between the WT and the transgenic lines could be observed.

Changes in the levels of free polyamines correlate with activation or repression of developmental response pathways such as ripening and senescence (Ioannidis et al., 2014; Mattoo & Handa, 2008; Sobieszczuk‐Nowicka, 2017; Sobieszczuk‐Nowicka et al., 2016). Thus, many of the phenotypical changes that we observed in the leaves of PMT‐RNAi and the PR50‐RNAi plants, for example, that the plants seemed to age slower and had more chlorophyll and smaller leaf mesophyll cells, are probably caused by the higher polyamine concentrations. The ODC inhibitor DMFO counteracted the elevated polyamine concentrations in the transgenic plants but could not totally reverse the effect of the transgenes as even in the DFMO‐treated plants polyamine content was still higher than in the WT plants. This might be due to not using a high enough concentration of DFMO; the 2 mM DFMO used in this study is a higher concentration than in similar studies (Fernández‐Crespo et al., 2016), however, the tobacco plants that we treated were large while other studies used small plants or seedlings. Furthermore, the tobacco plants were planted in soil to imitate cultivation in the field; it is not clear how much of the applied DFMO was actually taken up by the plants, therefore, the effective concentration of DFMO might have been much lower than 2 mM.

In accordance with the reduced polyamine concentrations, we also saw a changed phenotype in the DFMO‐treated transgenic plants; the leaf mesophyll cells were bigger than in the untreated transgenic plants; and PR50‐RNAi treated with DFMO no longer showed a significant difference in leaf mesophyll cell size in comparison to the WT.

The treatment of plants in the field with DFMO is not readily feasible, but if inhibition of polyamine biosynthesis is needed to restore the product quality of the low‐alkaloid varieties, then other treatment approaches could be explored. Oligogalacturonides have been shown to reduce the expression of polyamine biosynthesis‐related enzymes (Falasca et al., 2008), and such a treatment may have the additional benefit of further reducing the nicotine content of the plants (Martinez et al., 2020) and helping the plants to defend themselves against pathogens in the absence of nicotine (Falasca et al., 2008; Ferrari et al., 2013). Based on the preliminary data reported here from greenhouse experiments, additional research is needed to better understand leaf quality of low‐alkaloid tobacco lines grown in the field with such treatments.

## 4 EXPERIMENTAL PROCEDURES

### 4.1 Plant material and growth conditions

Seeds of *Nicotiana tabacum* L. cv. TN90 were obtained from the US *Nicotiana* Germplasm Collection at North Carolina State University. Transgenic TN90 PMT‐RNAi and TN90 PR50‐RNAi lines developed earlier were used in this study (Kudithipudi & Hayes, 2018). Transgenic, along with control seeds, were germinated in pots under greenhouse conditions at 27/23℃ day/night temperature and a 16‐hr photoperiod (~200 mmol/s m^−2^; *λ* = 400–700 nm) at 70% relative humidity. Five‐week‐old tobacco plantlets were transferred to 13 L pots with standard substrate (Einheitserde, Fröndenberg, Germany) and grown in the greenhouse for 4 additional weeks as previously described (Nölke et al., 2018). Plants were topped when 50% of the plants had at least one open flower. After topping, plants grew for additional 4 weeks until harvest. Suckers were removed manually three times a week. Tobacco leaves used for all analyses were collected at four time points: before flowering (6.5‐week‐old plants), just before topping, (9‐week‐old plants), 1 week post‐topping (10‐week‐old plants), and at harvest (13‐week‐old plants and 4 weeks post‐topping). Root samples used for polyamine and enzymatic analysis were collected at topping and harvest. An overview about the experimental design can be found in Table S1.

For treatment, 2 mM DFMO (Synchem Ug & Co. KG, Felsberg, Germany) was diluted in the same amount of water used for daily irrigation and applied to PMT‐RNAi plants every 4 hr at 9 a.m., 12 a.m., 3 p.m., and 6 p.m. three times per week instead of the drip irrigation system. Twelve plants per line were treated with DFMO. Treatment started at topping and lasted for 4 weeks until harvest. TN90 plants were used as controls.

### 4.2 Chlorophyll and glucose measurements

The chlorophyll content was determined by measuring leaf absorbance in the red and infrared regions using a SPAD‐502 Plus device (Minolta Camera Co.) as previously described (Nölke et al., 2018) from four randomly selected plants of each line. Measurements were done at different stages of development: before flowering (6.5‐week‐old plants and 2.5 weeks before topping), at topping, 1 WPT, and at harvest (30 days post‐topping). The total chlorophyll content was calculated as average of all measured leaf chlorophyll values per plant to minimize the influence of leaf position.

For glucose analysis, 35 g of tissue from leaf 11 (numbered from base of the plant) was collected before flowering, at topping, 1 week post‐topping, and at harvest. Four plants were analyzed at each time point. Leaf material was flash frozen in liquid nitrogen, ground, and incubated at 4℃ for 10 min with moderate shaking. The leaf extract was filtered through Miracloth and centrifuged at 16,000× *g* for 5 min at 4℃. Then, the supernatant was collected and used for glucose analysis using Quantofix system (Macherey&Nagel) according to the manufacturer's instructions.

### 4.3 Leaf cell microscopy

Four leaf discs (1 cm^2^) cut from leaf 15 from six different plants at different development stages (before flowering, at topping, 1 WPT, and at harvest) were mounted on slides and imaged using a Leica DM R microscope (Leica) with a 10x air objective. Images were imported into ImageJ and Adobe Photoshop CS5.1 software and the cells per unit area were counted using Count Tool in the Photoshop CS5.

### 4.4 Determination of ODC and ADC activities

To determine enzymatic activities, 500 mg of tobacco leaf or root tissue collected from four different plants at topping (leaf 19, roots) and at harvest (leaf 24, roots) was ground in 1 ml HEPES extraction buffer (100 mM HEPES, 2 mM dithiothreitol (DTT), 1 mM EDTA, pH 7.5) and 100 mg of polyvinylpyrrolidone was added during grinding. Following centrifugation (13,000× *g*, 10 min, 4℃), the enzyme activities were measured using an isotopic method as described by Capell et al. (1998) by measuring the release of ^14^CO_2_. L‐[1‐^14^C]Arg and L‐[1‐^14^C]Orn was used as radioactive substrates.

### 4.5 Polyamine extraction and analysis

For polyamine analysis, 150 mg of leaf or root material was harvested from plants grown in the greenhouse at different stages of development: before flowering (leaves 6 and 12, numbered from base), at topping (leaves 19 and 23 and roots), and at harvest (leaves 23 and 24 and roots). Samples were collected, after 4 hr of illumination and were flash frozen in liquid nitrogen. For time‐course monitoring of total polyamine content, six plants were used for collection of root samples from PMT‐RNAi plants and eight WT, PR50‐RNAi, and PMT‐RNAi plants for collection of leaf and/or root samples. For analysis of free and conjugated polyamines, four biological replicates were used. Extraction of free and conjugated polyamines was performed as previously described (Nölke et al., 2018). The dansilation of free and conjugated polyamines was carried out with dansyl chloride as described by Flores and Galston (1982). The dansylated polyamines were measured by LC‐MS/MS. All experiments were carried out on a 3200 QTRAP™ mass spectrometer (Sciex) coupled to an HPLC Agilent 1200 system as described before (Nölke et al., 2018).

### 4.6 Statistical analysis

Significant differences between the genotypes were determined by applying one‐way analysis of variance (ANOVA) followed by post‐hoc Bonferroni test using Excel software (Microsoft). Two‐tailed *t*‐tests were applied. A *p*‐value <.05 was considered statistically significant.

## CONFLICT OF INTEREST

M.L., J.F., C.K., Y.S., U.W., J.A.S., and D.X. were employed by Altria Client Services, and Altria Client Services provided funding for the research. All authors declare no conflict of interest.

## AUTHORS CONTRIBUTIONS

DX, UW, ML, and JAS conceived the original research plans; GN, DV, IC, MH, and HS performed the experiments; GN and HS designed and supervised the experiments; DV, IC and MH established and developed protocols; ML, JF, YS, CK, UW, DX, and JAS contributed reagents/materials/analysis tools; GN and HS analyzed and interpreted the data; GN, HS, SS, and ML wrote the article. All authors read and approved the final manuscript.

## Supporting information

Table S1
