# Supplementary material for: Impact of nicotine pathway downregulation on polyamine biosynthesis and leaf ripening in tobacco
Source: Plant Direct. 2021 May 27;5(5):e00329. doi: 10.1002/pld3.329 (PMC8156150; doi:10.1002/pld3.329)
Supplement: Supplementary file 1 — Table S1 [file PLD3-5-e00329-s001.pptx]

## Slide 1
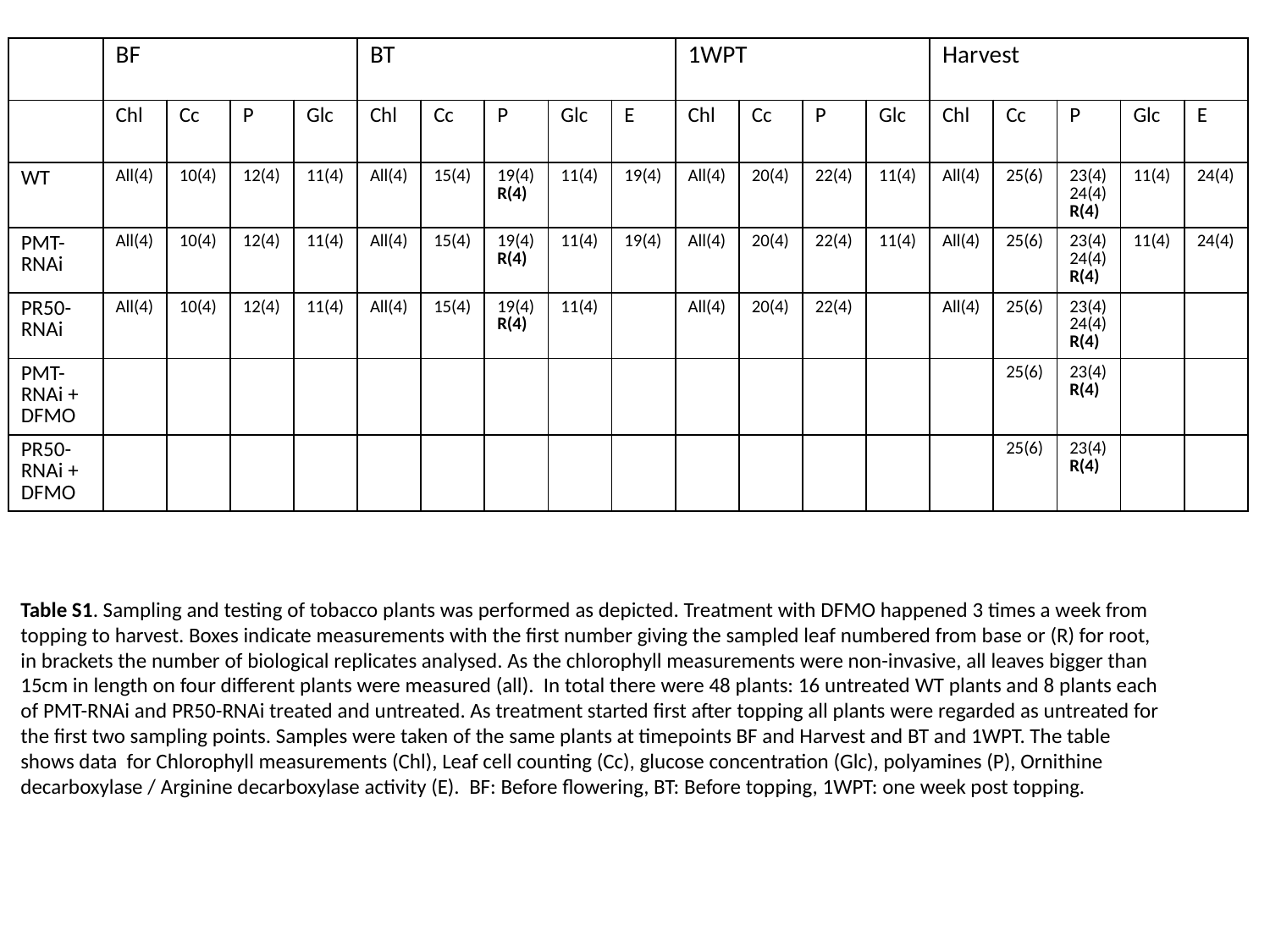

| | BF | | | | BT | | | | | 1WPT | | | | Harvest | | | | |
| --- | --- | --- | --- | --- | --- | --- | --- | --- | --- | --- | --- | --- | --- | --- | --- | --- | --- | --- |
| | Chl | Cc | P | Glc | Chl | Cc | P | Glc | E | Chl | Cc | P | Glc | Chl | Cc | P | Glc | E |
| WT | All(4) | 10(4) | 12(4) | 11(4) | All(4) | 15(4) | 19(4) R(4) | 11(4) | 19(4) | All(4) | 20(4) | 22(4) | 11(4) | All(4) | 25(6) | 23(4) 24(4) R(4) | 11(4) | 24(4) |
| PMT-RNAi | All(4) | 10(4) | 12(4) | 11(4) | All(4) | 15(4) | 19(4) R(4) | 11(4) | 19(4) | All(4) | 20(4) | 22(4) | 11(4) | All(4) | 25(6) | 23(4) 24(4) R(4) | 11(4) | 24(4) |
| PR50-RNAi | All(4) | 10(4) | 12(4) | 11(4) | All(4) | 15(4) | 19(4) R(4) | 11(4) | | All(4) | 20(4) | 22(4) | | All(4) | 25(6) | 23(4) 24(4) R(4) | | |
| PMT-RNAi + DFMO | | | | | | | | | | | | | | | 25(6) | 23(4) R(4) | | |
| PR50-RNAi + DFMO | | | | | | | | | | | | | | | 25(6) | 23(4) R(4) | | |
Table S1. Sampling and testing of tobacco plants was performed as depicted. Treatment with DFMO happened 3 times a week from topping to harvest. Boxes indicate measurements with the first number giving the sampled leaf numbered from base or (R) for root, in brackets the number of biological replicates analysed. As the chlorophyll measurements were non-invasive, all leaves bigger than 15cm in length on four different plants were measured (all). In total there were 48 plants: 16 untreated WT plants and 8 plants each of PMT-RNAi and PR50-RNAi treated and untreated. As treatment started first after topping all plants were regarded as untreated for the first two sampling points. Samples were taken of the same plants at timepoints BF and Harvest and BT and 1WPT. The table shows data for Chlorophyll measurements (Chl), Leaf cell counting (Cc), glucose concentration (Glc), polyamines (P), Ornithine decarboxylase / Arginine decarboxylase activity (E). BF: Before flowering, BT: Before topping, 1WPT: one week post topping.
